# The impact of new psychosocial stressors on the mental health of young people: Results from a national multicentric study in italy

**DOI:** 10.1192/j.eurpsy.2021.210

**Published:** 2021-08-13

**Authors:** G. Sampogna

**Affiliations:** Department Of Psychiatry, University of Campania “Luigi Vanvitelli”, Naples, Italy

## Abstract

**Abstract Body:**

The COVID-19 pandemic with the related containment measures is having a negative impact on the mental health of the general population worldwide. This event has been described as a new form of trauma, which is influencing not only physical and mental health, but also the society as a whole. Among Western countries, Italy has been one of the first severely hit by the pandemic in terms of number of cases and mortality rates. In March, 2020, strictly restrictive measures has been issued in order to contain the spread of the disease. This period has been known as “Phase one” of the national health emergency, where all not necessary activities were closed, almost 30,000 people died and almost 100,000 people were home-isolated. In this context, the COvid Mental hEalth Trial (COMET) network, including ten university Italian sites and the National Institute of Health, has been established in order to promote a national online survey for assessing the impact of lockdown measures on the mental health of the Italian general population. In the COMET survey, it has been included a specific focus on young people, which are expected to be the most vulnerable to the consequences of the pandemic and of the strict containment measures. Findings from this study can be useful to inform national and international associations on the importance to provide adequate support to the mental health of the young people.

**Disclosure:**

No significant relationships.

